# Development and validation of a nomogram for predicting cancer-specific survival in small-bowel adenocarcinoma patients using the SEER database

**DOI:** 10.1186/s12957-024-03438-x

**Published:** 2024-06-07

**Authors:** Duogang Xu, Yulei He, Changkang Liao, Jing Tan

**Affiliations:** 1 Department of General Surgery, Yan’an Hospital Affiliated to Kunming Medical University, Kunming, China; 2 Key Laboratory of Tumor Immunological Prevention and Treatment of Yunnan Province, Yunnan University of Chinese Medicine, Kunming, China; 3 The First School of Clinical Medicine, Yunnan University of Chinese Medicine, Kunming, China

**Keywords:** Small bowel adenocarcinoma, Cancer-specific survival, SEER database, Nomogram, Neoplasm staging

## Abstract

**Background:**

Small bowel adenocarcinoma (SBA) is a rare gastrointestinal malignancy forwhich survival is hampered by late diagnosis, complex responses to treatment, and poor prognosis. Accurate prognostic tools are crucial for optimizing treatment strategies and improving patient outcomes. This study aimed to develop and validate a nomogram based on the Surveillance, Epidemiology, and End Results (SEER) database to predict cancer-specific survival (CSS) in patients with SBA and compare it to traditional American Joint Committee on Cancer (AJCC) staging.

**Methods:**

We analyzed data from 2,064 patients diagnosed with SBA between 2010 and 2020 from the SEER database. Patients were randomly assigned to training and validation cohorts (7:3 ratio). Kaplan‒Meier survival analysis, Cox multivariate regression, and nomograms were constructed for analysis of 3-year and 5-year CSS. The performance of the nomograms was evaluated using Harrell’s concordance index (C-index), the area under the receiver operating characteristic (ROC) curve, calibration curves, decision curve analysis (DCA), net reclassification improvement (NRI), and integrated discrimination improvement (IDI).

**Results:**

Multivariate Cox regression identified sex, age at diagnosis, marital status, tumor site, pathological grade, T stage, N stage, M stage, surgery, retrieval of regional lymph nodes (RORLN), and chemotherapy as independent covariates associated with CSS. In both the training and validation cohorts, the developed nomograms demonstrated superior performance to that of the AJCC staging system, with C-indices of 0.764 and 0.759, respectively. The area under the curve (AUC) values obtained by ROC analysis for 3-year and 5-year CSS prediction significantly surpassed those of the AJCC model. The nomograms were validated using calibration and decision curves, confirming their clinical utility and superior predictive accuracy. The NRI and IDI indicated the enhanced predictive capability of the nomogram model.

**Conclusion:**

The SEER-based nomogram offers a significantly superior ability to predict CSS in SBA patients, supporting its potential application in clinical decision-making and personalized approaches to managing SBA to improve survival outcomes.

## Introduction

The small intestine, which is composed of the duodenum, jejunum, and ileum, plays a crucial role in the gastrointestinal tract, accounting for more than 75% of its length and 90% of its mucosal surface area [1–3]. Despite its extensive surface area, malignancies of the small intestine are exceedingly rare, constituting fewer than 5% of all gastrointestinal cancers [4]. Like testicular cancer and Hodgkin’s lymphoma, tumors of the small intestine are also rare, with thousands of new cases annually reported in the United States and Europe [5]. Small bowel adenocarcinoma (SBA) accounts for approximately 30–40% of small bowel tumors. Owing to its low incidence, typically late diagnosis, and poor prognosis, the five-year survival rate for advanced SBA patients often falls below 50%, posing unique challenges for treatment [6]. Despite complex therapies, the prognosis for patients with SBA remains poor, with a low median survival [7]. Therefore, accurate strategies for the prognostic analysis of SBA patients are urgently needed to tailor individual treatments and monitoring methods.

Clinical treatment approaches for SBA are often similar to those for colorectal cancer (CRC) owing to their anatomical proximity and shared molecular characteristics, allowing the use of similar surgical and adjuvant therapies [8]. However, due to significant differences in their genetic profiles and molecular characteristics, as well as in the tumor microenvironment, such as a lower bacterial load and unique immunological features, SBA may require distinct prognostic models and treatment strategies [9]. Current prognostic assessments primarily rely on the American Joint Committee on Cancer (AJCC) TNM staging system, which, despite its widespread use, may not fully reflect the clinicopathological features of SBA [10]. This underscores the need for more targeted prognostic tools.

To address these challenges, our research focused on the application of nomograms—visual representations of complex mathematical models—to enhance the accuracy of prognostic assessments for SBA patients. These tools integrate various clinical and pathological factors, enabling personalized predictions of survival outcomes [11–13]. Due to the rarity of adenocarcinoma of the small intestine, it is difficult to collect enough specimens for analysis in the clinic. We utilized large-scale databases, such as the Surveillance, Epidemiology, and End Results (SEER) database, to construct predictive models for SBA, facilitating more effective clinical decision-making.

This article presents a newly constructed nomogram based on SEER data aimed at predicting cancer-specific survival (CSS) in SBA patients. By comparing the effectiveness of this nomogram with that of traditional staging systems and conducting subgroup risk and treatment analyses, this study contributes to the optimization of diagnostic and treatment strategies for SBA, ultimately aiming to improve patient outcomes. The article is structured according to the TRIPOD statement checklist (10.21037/apm-21-600).

## Materials and methods

### Patient selection

**Fig. 1:**
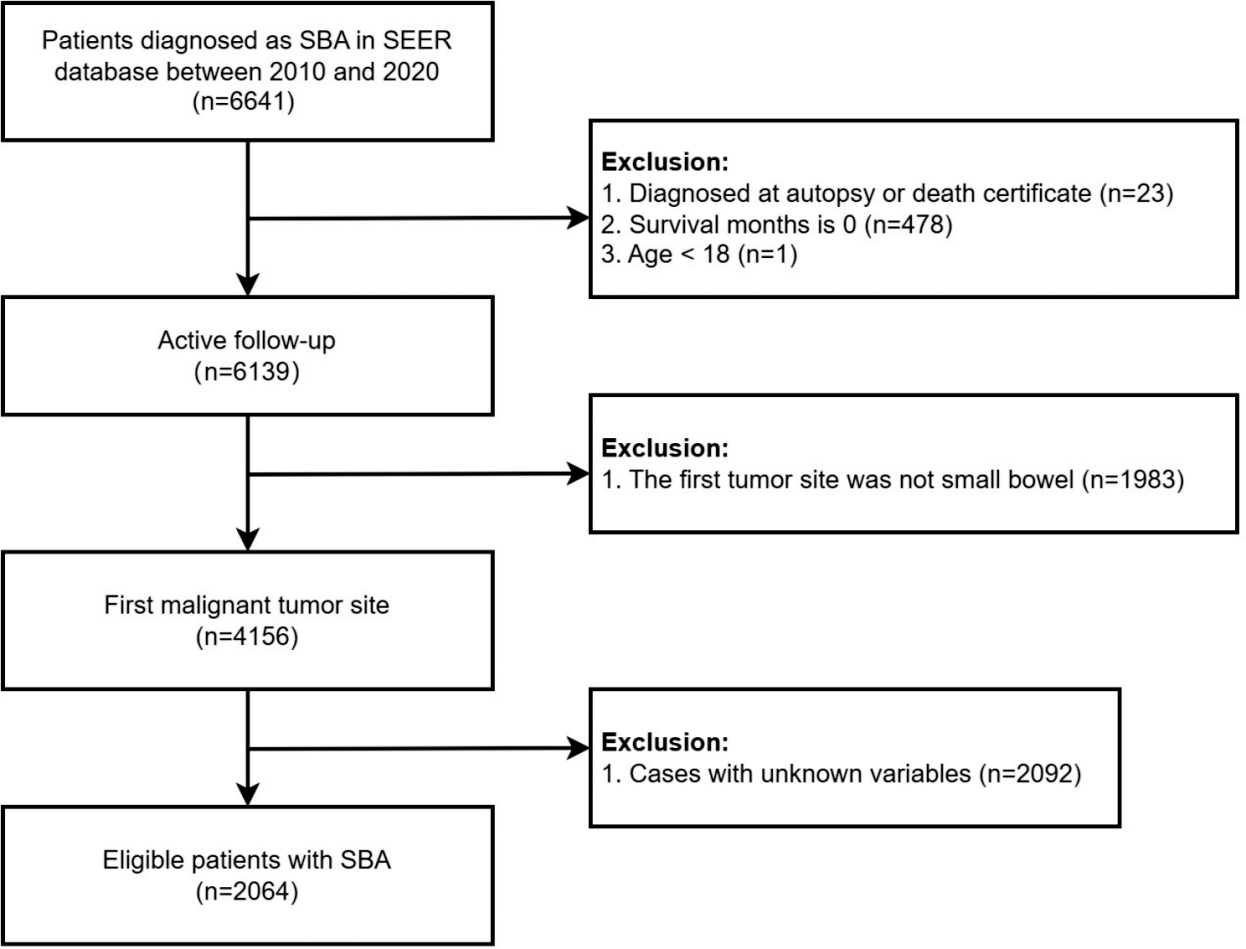
Flow chart for the selection of eligible patients with adenocarcinoma of the small bowel

The SEER database of the National Cancer Institute was utilized as the data source for this population-based study. Approximately 34.6% of the U.S. population is covered by the SEER database, which collects cancer incidence information from 18 cancer registries and includes data on patient demographics, tumor characteristics, treatment, and survival for all incident cases [14]. After obtaining permission to access the SEER research files, data for 6,641 patients diagnosed with SBA between 2010 and 2020 were extracted. Patients were identified using the SEER variables “Site Recode ICD-O-3/WHO 2008 classification” for the small intestine and “Histology Recode-Broad Group” for histology codes 8140–8389. Survival information was obtained from the “SEER Cause Specific Death Classification” and “Survival Months” codes. CSS was defined as the time from SBA diagnosis to death, specifically from SBA. The exclusion criteria were as follows: (1) diagnosis by autopsy or death certificate; (2) survival months = 0; (3) under 18 years of age; (4) the patient’s first primary tumor was not SBA; and (5) incomplete clinicopathological information. The exclusion process is illustrated in Fig. 1. Ultimately, 2,064 patients were included in the study. Random grouping was then performed using R software at a ratio of 7:3 (training group, *n* = 1,444; validation group, *n* = 620).

### Study variables and endpoints

The study collected data on variables including sex, age at diagnosis, marital status, race, tumor site, histologic grade, TNM (tumor, node, metastasis) stage, surgical intervention, retrieval of regional lymph nodes (RORLN), radiation therapy, and chemotherapy. The facial categories were classified as White, Black, or Other. The tumor sites were divided into the duodenum, jejunum, ileum, and other/unspecified sites. Tumors were classified into four grades: well differentiated (grade I), moderately differentiated (grade II), poorly differentiated (grade III), and undifferentiated (grade IV). T stages were categorized as T1–2, T3–4, or Tx; the N stages were categorized as N0, N1–N2, or Nx; and the M stages were categorized as M0 or M1. Surgical interventions were classified as none, radical, or palliative. Radical surgery was defined as the simultaneous resection of both the primary and metastatic tumors in a single procedure. Palliative surgery was defined as partial resection of the primary tumor, with or without the resection of metastatic tumors. The RORLNs were categorized into 0, 1–3, and ≥ 4 nodes. In the training group, univariate Cox regression analysis was used to screen for prognostic factors. To assess whether these factors could serve as independent prognostic indicators, multivariate Cox regression analysis was subsequently employed to confirm the independent prognostic factors and their effects on CSS, represented by hazard ratios (HRs). Preliminary prognostic factors (those with a P value < 0.05 in univariate analysis) were included in the multivariate Cox regression model for analysis. The endpoint of the study was CSS, with analyses focused on 3-year and 5-year CSS rates.

### Statistical analysis

The baseline clinicopathological characteristics of the training and validation cohorts were assessed using the chi-square test to determine baseline differences, if any. Kaplan‒Meier survival analysis was used to assess the associations between various variables and CSS. The significance of differences in survival curves was determined using log-rank tests. Multicollinearity among independent variables was addressed by applying a bidirectional stepwise regression selection method in the Cox regression model. Both univariate and multivariate Cox regression analyses, which were used to develop nomograms for 3-year and 5-year CSS, respectively, were performed to evaluate all variables and to identify independent prognostic factors.

The predictive performance of the nomograms was measured using Harrell’s concordance index (C-index) [15] and assessed through calibration curves. A higher C-index indicates better discriminative ability among patients with different survival outcomes. The predictive accuracy of the nomograms for 3-year and 5-year survival rates was evaluated and compared using the area under the receiver operating characteristic (ROC) curve. Improvements of the new predictive model over the AJCC staging system were determined using the net reclassification improvement (NRI) and integrated discrimination improvement (IDI) in both the training and validation cohorts. Decision curve analysis (DCA) was employed to test the clinical utility of the predictive model [16]. A two-tailed probability value of *P* < 0.05 was considered to indicate statistical significance. X-tile software (version 3.6.1, Yale School of Medicine, New Haven, CT, USA) was used to determine the optimal cutoff values and for risk stratification of patients. All analyses were conducted using R software (version 4.3.2; http://www.r-project.org).

## Results

### Clinical and pathological characteristics of patients

This study included data for 2,064 patients from the SEER database who met the eligibility criteria. The demographic and clinicopathological characteristics of the overall cohort are presented in Table 1. No statistically significant differences were observed between the training and validation groups. Overall, the majority of the patients were male, accounting for 1,132 patients (54.8%). The most represented age group was ≥ 70 years, comprising 744 patients (36%). The majority were married, totaling 1,253 patients (60.7%). White patients constituted the largest racial group, with 1,531 patients (74.18%). The duodenum was the most common site of SBA, with 1,082 cases (52.4%). Stages T3–T4 were predominant, involving 1,602 patients (77.6%), with 1,023 patients (49.6%) at the N0 stage and 1,492 patients (72.3%) showing no metastasis. Of these patients, 1,651 (80%) underwent surgical treatment, and 1,130 (53.4%) received chemotherapy. Additionally, 1,278 patients (61.9%) had a regional lymph node resection count ≥ 4.

**Table 1:** Patient baseline characteristics

Variables	Total (*n* = 2064)	Training group (*n* = 1444)	Validation group (*n* = 620)	*P* value
Gender, n (%)				0.769
Male	1132 (54.84)	795 (55.06)	337 (54.35)	
Female	932 (45.16)	649 (44.94)	283 (45.65)	
Age, n (%)				0.361
≤ 50	310 (15.02)	220 (15.24)	90 (14.52)	
51–60	445 (21.56)	296 (20.50)	149 (24.03)	
61–70	565 (27.37)	400 (27.70)	165 (26.61)	
≥ 70	744 (36.05)	528 (36.57)	216 (34.84)	
Marital status, n (%)				0.067
Married	1253 (60.71)	858 (59.42)	395 (63.71)	
Unmarried	811 (39.29)	586 (40.58)	225 (36.29)	
Race, n (%)				0.947
White	1531 (74.18)	1069 (74.03)	462 (74.52)	
Black	374 (18.12)	262 (18.14)	112 (18.06)	
Other	159 (7.7)	113 (7.83)	46 (7.42)	
Tumor site, n (%)				0.851
Duodenum	1082 (52.42)	761 (52.70)	321 (51.77)	
Jejunum	374 (18.12)	259 (17.94)	115 (18.55)	
Ileum	345 (16.72)	245 (16.97)	100 (16.13)	
Other/NOS^*^	263 (12.74)	179 (12.40)	84 (13.55)	
Pathological grade, n (%)				0.377
I	204 (9.88)	134 (9.28)	70 (11.29)	
II	1054 (51.07)	740 (51.25)	314 (50.65)	
III	777 (37.65)	552 (38.23)	225 (36.29)	
IV	29 (1.41)	18 (1.25)	11 (1.77)	
T stage, n (%)				0.999
T1-T2	343 (16.62)	240 (16.62)	103 (16.61)	
T3-T4	1602 (77.62)	1121 (77.63)	481 (77.58)	
Tx	119 (5.77)	83 (5.75)	36 (5.81)	
N stage, n (%)				0.999
N0	1023 (49.56)	709 (49.10)	314 (50.65)	
N1-N2	988 (47.87)	699 (48.41)	289 (46.61)	
Nx	53 (2.57)	36 (2.49)	17 (2.74)	
M stage, n (%)				0.654
M0	1492 (72.29)	1048 (72.58)	444 (71.61)	
M1	572 (27.71)	396 (27.42)	176 (28.39)	
Surgery, n (%)				0.388
No	413 (20.01)	293 (20.29)	120 (19.35)	
Palliative surgery	1220 (59.11)	840 (58.17)	380 (61.29)	
Radical surgery	431 (20.88)	311 (21.54)	120 (19.35)	
RORLN^*^, n (%)				0.386
0	594 (28.78)	419 (29.02)	175 (28.23)	
1–3	192 (9.30)	126 (8.73)	66 (10.65)	
≥ 4	1278 (61.92)	899 (62.26)	379 (61.13)	
Radiation, n (%)				0.910
No	1882 (91.18)	1316 (91.14)	566 (91.29)	
Yes	182 (8.82)	128 (8.86)	54 (8.71)	
Chemotherapy, n (%)				0.608
No	961 (46.56)	667 (46.19)	294 (47.42)	
Yes	1103 (53.44)	777 (53.81)	326 (52.58)	

^*^ NOS: Not otherwise specified; RORLN: Retrieval of regional lymph nodes

### Survival analysis of variables

**Fig. 2:**
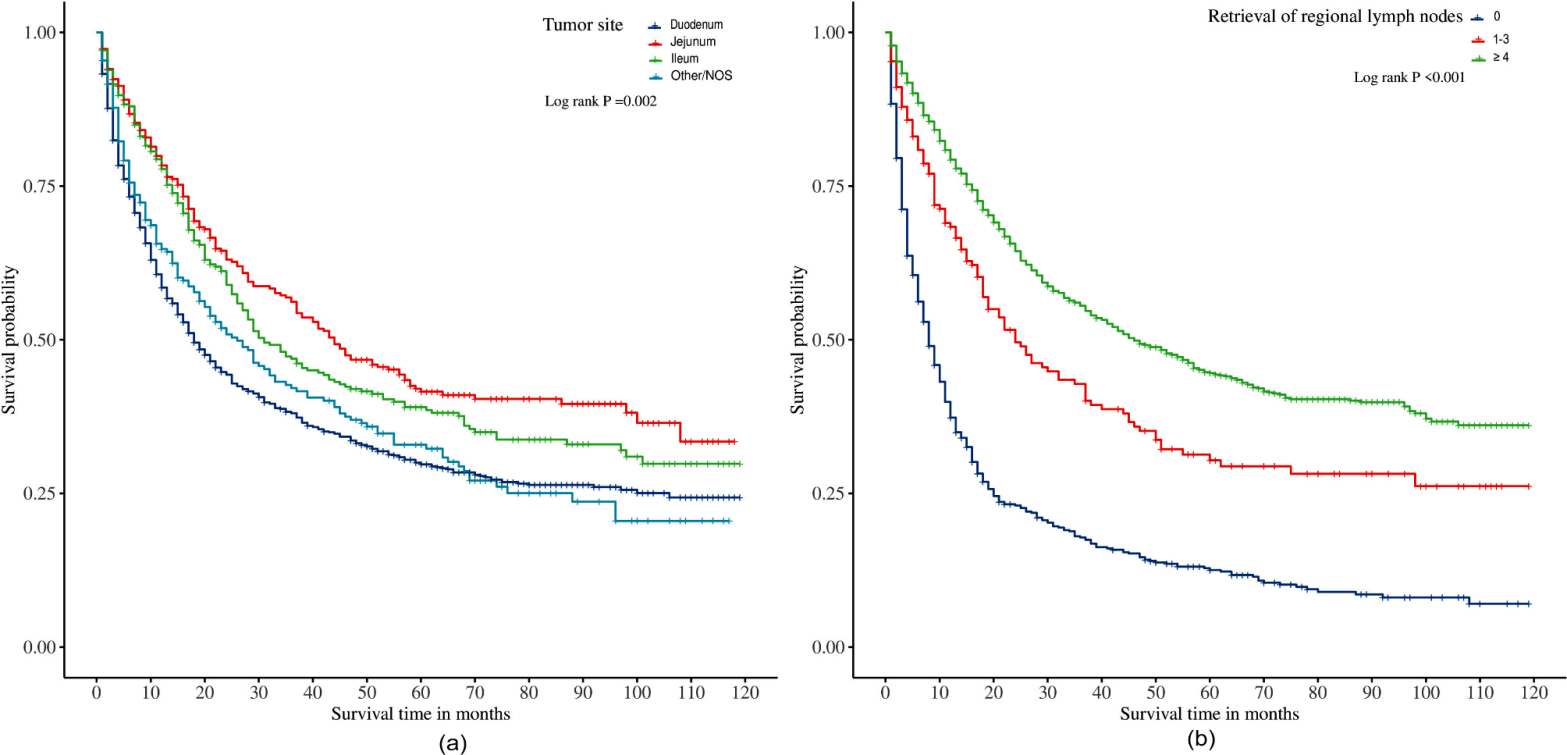
Effect of variables on CSS in patients with adenocarcinoma of the small bowel. (**A**) Tumor site; (**B**) Retrieval of regional lymph nodes

To evaluate the impact of tumor location and the number of regional lymph node resections on the CSS of patients with SBA, Kaplan‒Meier survival analysis was conducted for all patients. As illustrated in Fig. 2A, there was a statistically significant difference in CSS for patients with different tumor sites (*P* < 0.001). Patients with SBAs located in the jejunum and ileum exhibited the highest survival rates, while those with tumors in other unspecified sites and the duodenum had poorer survival rates. Furthermore, Kaplan‒Meier survival analysis for regional lymph node resection demonstrated that a greater number of resected lymph nodes was associated with higher survival rates among SBA patients, with significant differences in survival observed across groups (*P* < 0.001) (Fig. 2B).

### Construction of nomograms

**Fig. 3:**
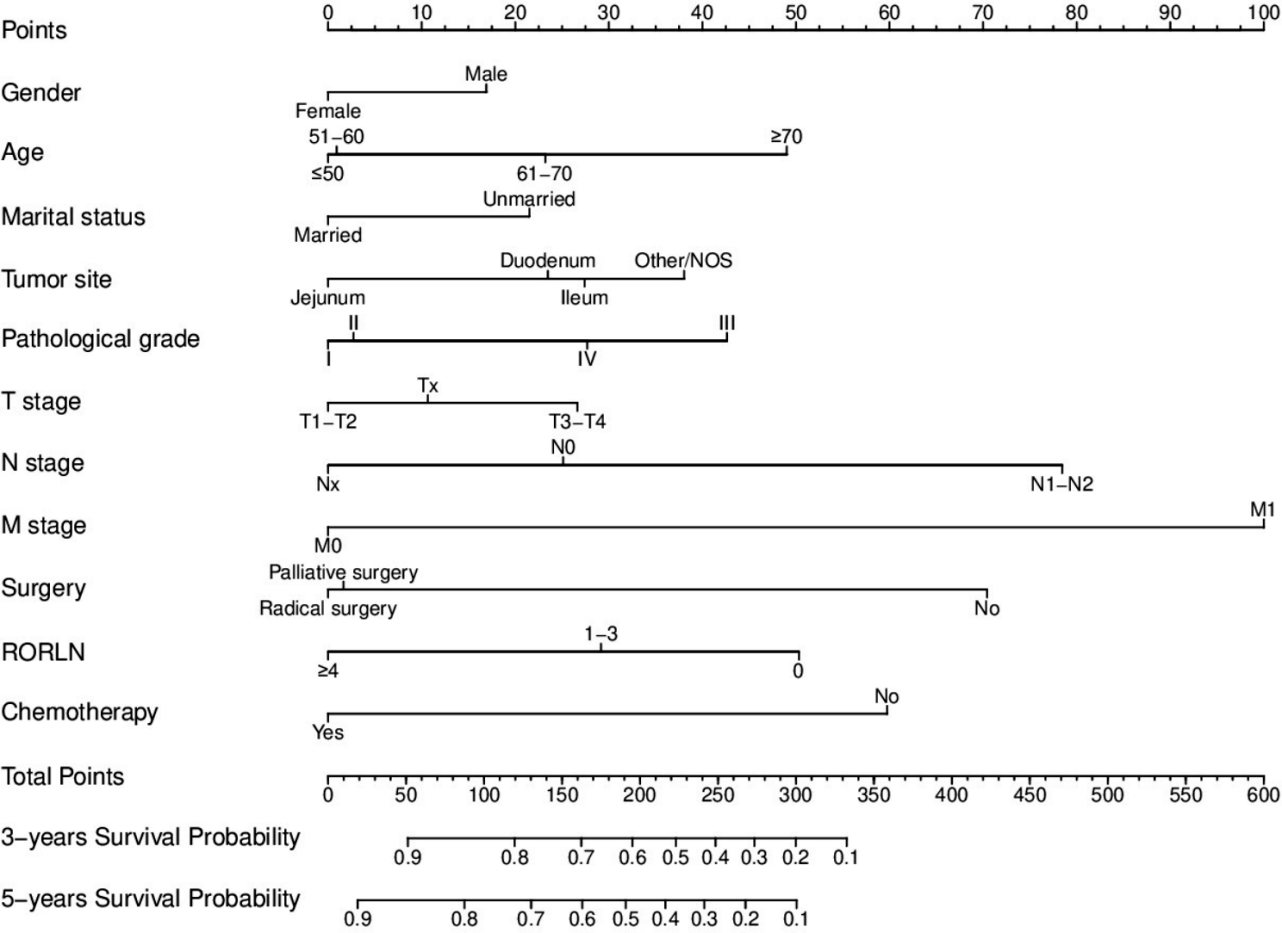
Nomogram for predicting the 3- and 5-year CSS of patients with SBA

Eleven variables were confirmed to be independent predictors of CSS, namely, sex, age, marital status, tumor location, pathological grade, T stage, N stage, M stage, surgical intervention, RORLN, and chemotherapy (Table 2), all of which showed significant associations (P value < 0.05 in multivariate analysis). Using these 11 statistically significant variables, nomograms were created to predict 3-year and 5-year CSS (Fig. 3). By summing the scores associated with each variable and projecting the total score onto the baseline scale, the estimated probabilities for 3-year and 5-year CSS could be calculated. The nomograms indicate that the presence of metastasis has the greatest impact on prognosis, with surgery, N stage, and chemotherapy also contributing significantly to patient outcomes.

**Table 2:** Univariate and multivariate analyses of cancer-specific survival for the training cohort

Variables	Univariate HR (95% CI)	*P* value	Multivariate HR (95%CI)	*P* value
Gender				
Male	Reference		Reference	
Female	0.87 (0.76-1.00)	0.042	0.83 (0.72–0.95)	0.009
Age				
≤ 50	Reference		Reference	
51–60	1.22 (0.96–1.55)	0.110	1.02 (0.80–1.31)	0.870
61–70	1.28 (1.01–1.62)	0.037	1.31 (1.03–1.66)	0.026
≥ 70	1.97 (1.58–2.45)	<0.001	1.75 (1.39–2.20)	<0.001
Marital status				
Married	Reference		Reference	
Unmarried	1.25 (1.09–1.43)	0.002	1.24 (1.07–1.44)	0.004
Race				
White	Reference		Reference	
Black	1.12 (0.95–1.32)	0.190	1.14 (0.95–1.36)	0.149
Other	1.06 (0.82–1.38)	0.664	1.04 (0.80–1.36)	0.766
Tumor site				
Duodenum	Reference		Reference	
Jejunum	0.63 (0.52–0.77)	<0.001	0.78 (0.62–0.98)	0.032
Ileum	0.75 (0.62–0.91)	0.003	1.06 (0.85–1.33)	0.586
Other/NOS^*^	0.89 (0.72–1.11)	0.300	1.18 (0.93–1.50)	0.163
Pathological grade				
I	Reference		Reference	
II	1.18 (0.92–1.52)	0.198	1.02 (0.78–1.31)	0.909
III	1.87 (1.45–2.42)	<0.001	1.59 (1.22–2.07)	0.001
IV	2.12 (1.23–3.65)	0.007	1.39 (0.79–2.43)	0.254
T stage				
T1-T2	Reference		Reference	
T3-T4	0.98 (0.81–1.18)	0.820	1.34 (1.09–1.64)	0.005
Tx	3.91 (2.97–5.15)	<0.001	1.13 (0.84–1.53)	0.417
N stage				
N0	Reference		Reference	
N1-N2	1.41 (1.23–1.61)	<0.001	1.80 (1.53–2.11)	<0.001
Nx	3.39 (2.36–4.87)	<0.001	0.77 (0.51–1.15)	0.202
M stage				
M0	Reference		Reference	
M1	3.85 (3.34–4.44)	<0.001	3.00 (2.50–3.61)	<0.001
Surgery				
No	Reference		Reference	
Palliative surgery	0.23 (0.19–0.27)	<0.001	0.47 (0.36–0.61)	<0.001
Radical surgery	0.22 (0.18–0.27)	<0.001	0.46 (0.34–0.62)	<0.001
RORLN^*^				
0	Reference		Reference	
1–3	0.46 (0.36–0.59)	<0.001	0.81 (0.61–1.08)	0.147
≥ 4	0.32 (0.28–0.37)	<0.001	0.59 (0.46–0.74)	<0.001
Radiation				
No	Reference		Reference	
Yes	1.18 (0.95–1.48)	0.136	1.15 (0.90–1.45)	0.260
Chemotherapy				
No	Reference		Reference	
Yes	0.86 (0.75–0.98)	0.027	0.52 (0.44–0.61)	<0.001

^*^ NOS: Not otherwise specified; RORLN: Retrieval of regional lymph nodes

### Validation of the nomograms

To determine the discriminative ability and calibration of the nomograms, this study employed several methods, including the ROC curve, Harrell’s C-index, and calibration curves.

**Fig. 4:**
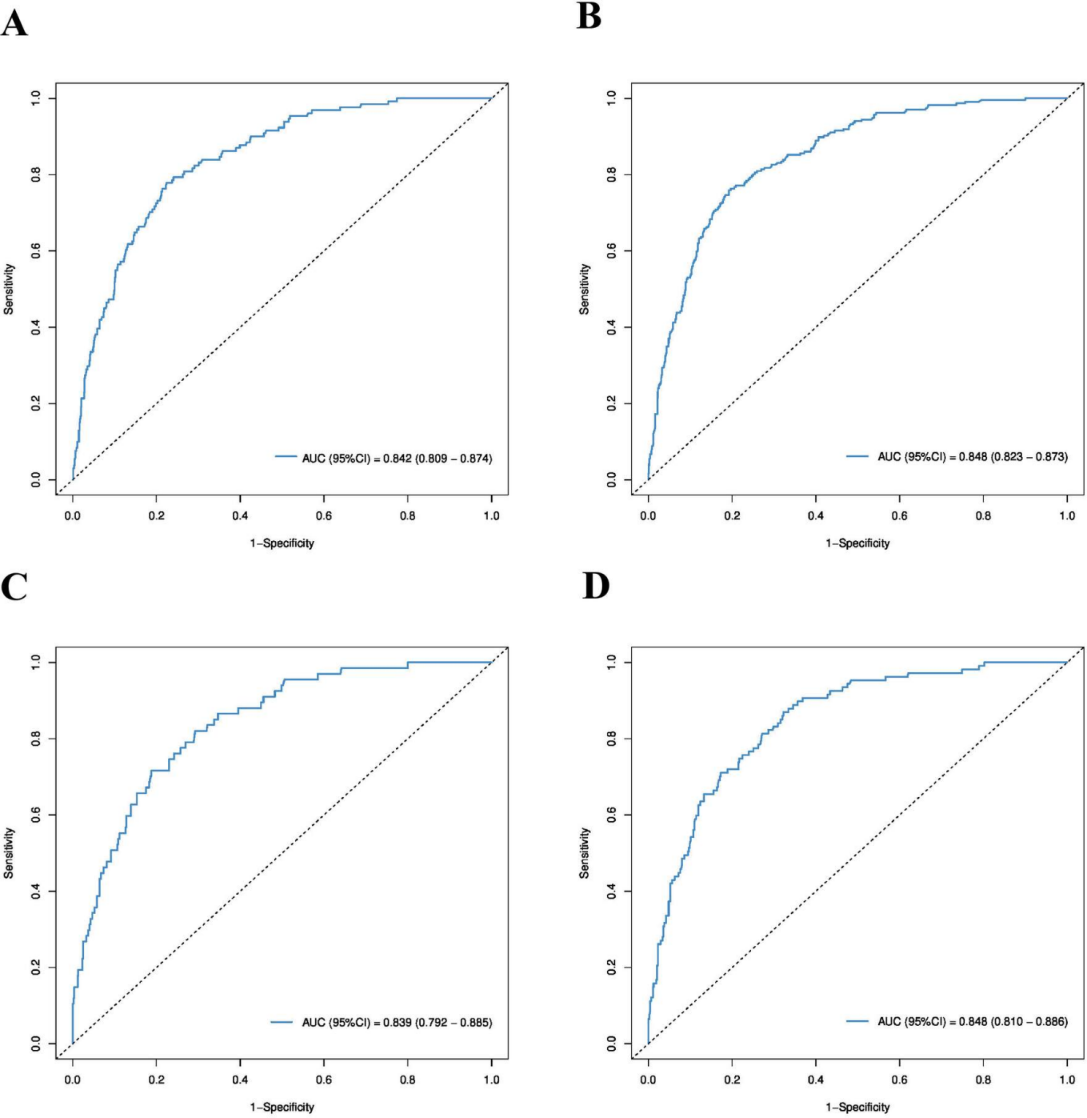
Comparison of the ROC curve of the nomogram for the prediction of CSS in the training group (**A**: 3 years; **B**: 5 years) and the validation group (**C**: 3 years; **D**: 5 years)

**Fig. 5:**
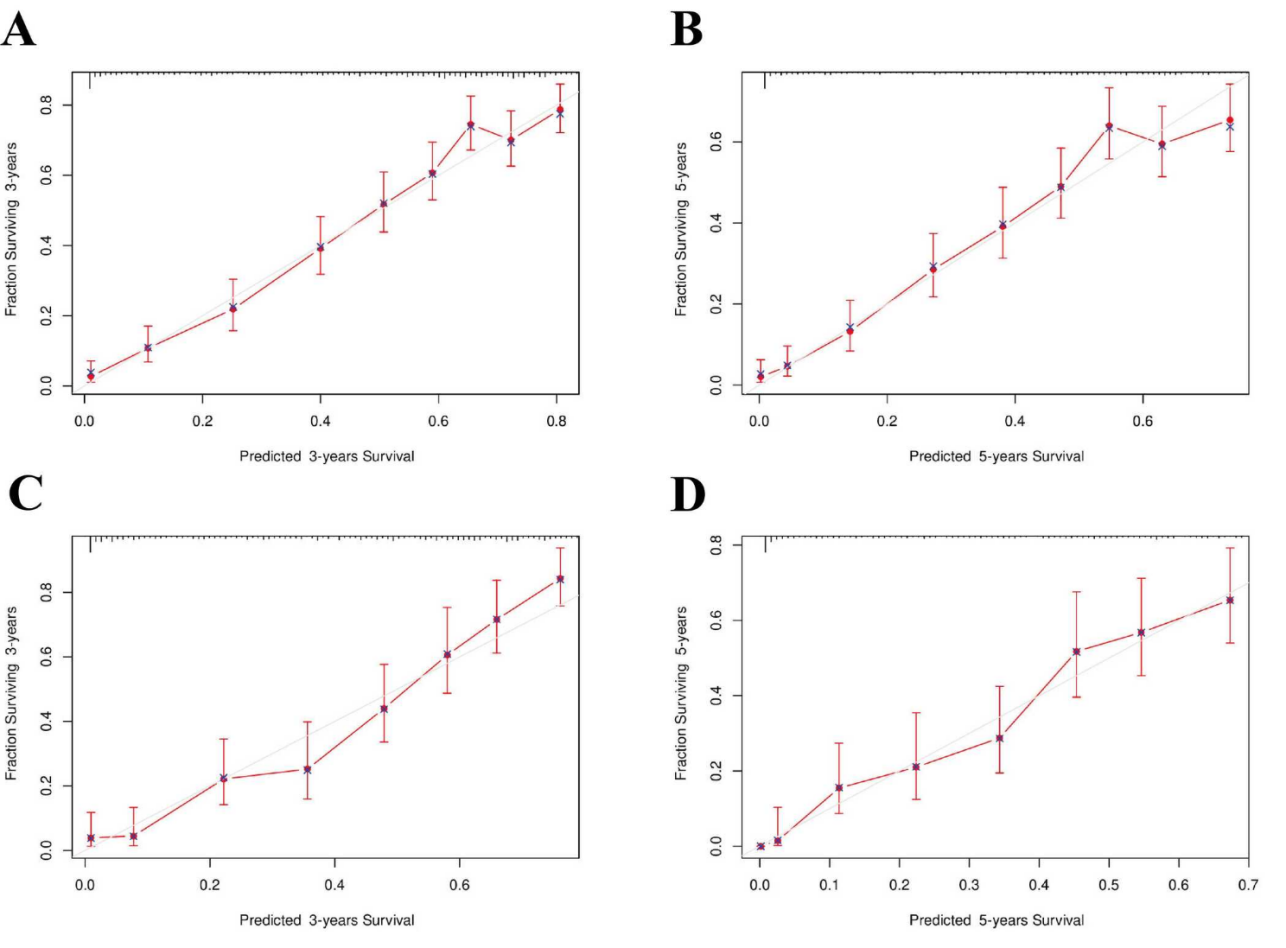
The calibration of the nomograms using the training group and validation group. (**A**) 3-year CSS and (**B**) 5-year CSS according to the training group. (**C**) 3-year CSS and (**D**) 5-year CSS according to the training group

For the CSS nomograms, the C-indexes for the training and validation groups were 0.764 (95% CI, 0.749–0.779) and 0.759 (95% CI, 0.736–0.783), respectively, which were greater than those for the AJCC staging CSS (0.663 (95% CI, 0.645–0.682) in the training cohort and 0.669 (95% CI, 0.641–0.695) in the validation cohort). This indicates the improved discriminative ability of the nomograms compared with that of the AJCC staging system. Time-dependent ROC analysis for 3-year and 5-year intervals confirmed that, compared with the AJCC staging system, the nomograms exhibited greater sensitivity and specificity in predicting the prognosis of SBA patients. The area under the curve (AUC) values for 3-year and 5-year CSS in the training group were 0.842 and 0.848, respectively, compared to 0.716 and 0.724 for AJCC staging. In the validation group, the AUC values for 3-year and 5-year CSS were 0.839 and 0.848, respectively, compared to 0.754 and 0.752 for AJCC staging. The ROC curves for 3-year and 5-year CSS in both the training and validation groups, shown in Fig. 4, illustrate the superior survival prediction capability of the nomograms over the AJCC staging system. Calibration of the nomograms was performed using bootstrap resampling with 1000 samples. The calibration curves for the predicted 3-year and 5-year CSS closely aligned with the actual observations, especially in the training cohort (Fig. 5). This indicates that the CSS nomograms were well validated, demonstrating their reliability and accuracy in predicting outcomes for SBA patients.

**Fig. 6:**
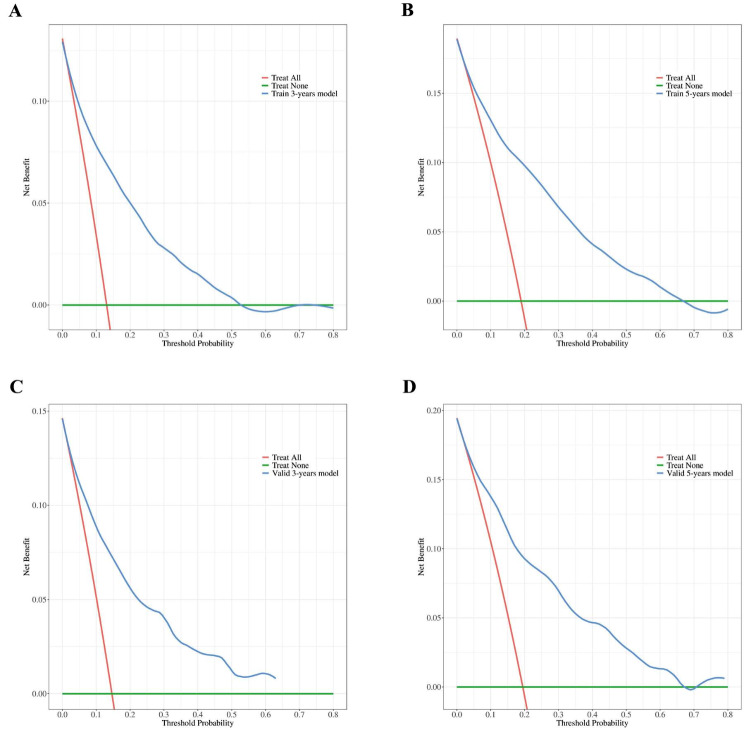
The DCA of the nomograms using the training group and validation group. (**A**) 3-year CSS and (**B**) 5-year CSS according to the training group. (**C**) 3-year CSS and (**D**) 5-year CSS according to the training group

The DCA for 3-year and 5-year survival was performed with the threshold probability on the x-axis and the net benefit on the y-axis. The curves demonstrate that the predictive model provides a significant positive net benefit across both the training and validation cohorts in terms of assessing the risk of death. This indicates that the nomograms have good clinical value in predicting 3-year and 5-year CSS. The DCA, as illustrated in Fig. 6, showed that using the nomograms to predict survival yields greater benefits than treating all or no patients across a range of reasonable threshold probabilities. This enhancement in decision-making underscores the practical utility of the nomograms in clinical settings, allowing healthcare providers to make more informed decisions based on patients’ specific risk profiles.

To further assess the improvement in the performance of the nomogram model over that of the traditional AJCC staging model, the NRI and IDI were calculated for 3-year and 5-year CSS. The NRIs for the nomogram model were 0.416 (95% CI = 0.337–0.506, *P* < 0.001) for the 3-year CSS rate and 0.372 (95% CI = 0.285–0.431, *P* < 0.001) for the 5-year CSS rate. These values indicate that the nomogram model significantly improved the classification of patient outcomes compared with the AJCC staging model. Similarly, the IDI values were 0.174 (95% CI = 0.091–0.236, *P* < 0.001) for the 3-year CSS rate and 0.138 (95% CI = 0.077–0.201, *P* < 0.001) for the 5-year CSS rate. These results show a substantial improvement in the discriminatory power of the nomogram model relative to the AJCC staging model for both the 3-year and 5-year intervals. These metrics (NRI and IDI) demonstrate that the nomogram provides a significantly enhanced ability to predict CSS, confirming its superiority over traditional staging systems. These findings support the clinical utility of the nomogram in providing more accurate prognostic information for patients with SBA, thereby aiding in better individualized patient management.

### Risk group stratification

**Fig. 7:**
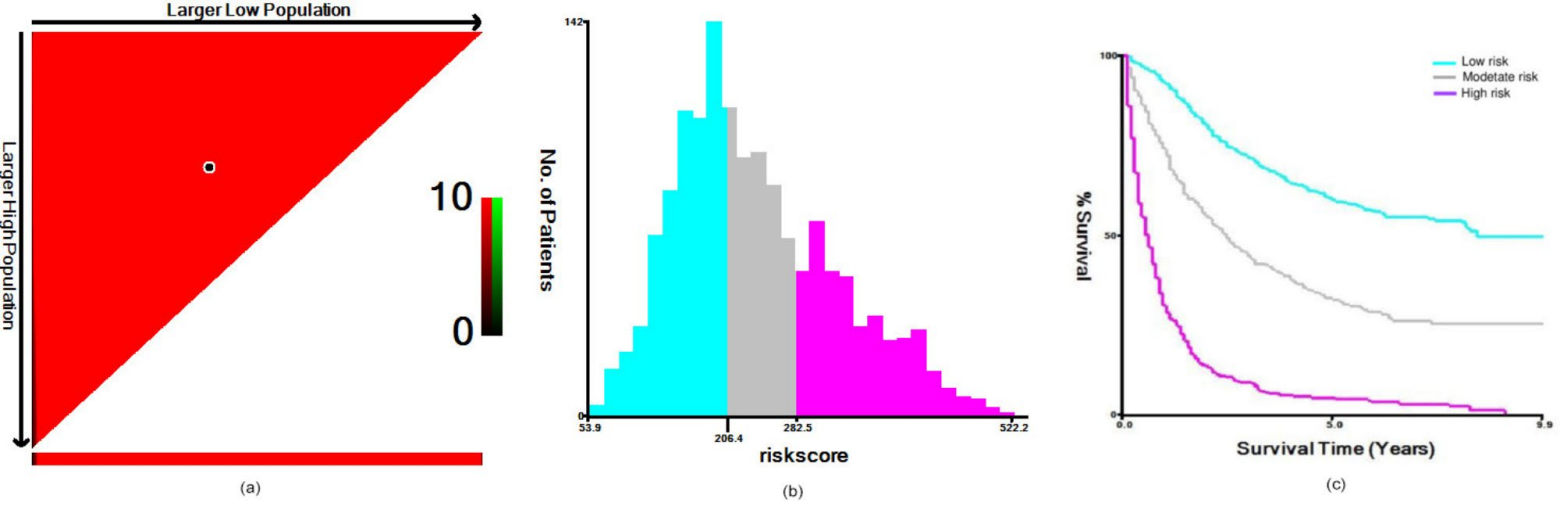
X-tile analysis was used to determine the optimal threshold for the total score for categorizing patients with adenocarcinoma of the small bowel into three risk subgroups. (**A**) Selection points for optimal cutoff values. (**B**) Histograms of the risk subgroups and the corresponding total scores. (**C**) Kaplan‒Meier overall survival curves for the three risk subgroups

Based on the established nomograms, the prognostic scores for all variables were calculated for each patient in the study. The optimal cutoff values for these total scores were determined using X-tile software. Based on the critical values from the CSS nomograms, patients with SBAs were stratified into three risk groups: a low-risk group (score ≤ 206), a moderate-risk group (score > 206 and ≤ 282), and a high-risk group (score > 282). Kaplan‒Meier analysis and the log-rank test revealed statistically significant differences in survival among the three groups (Fig. 7). This stratification allows clinicians to better understand the prognosis of SBA patients based on their risk category, facilitating more tailored and potentially more effective therapeutic strategies.

**Fig. 8:**
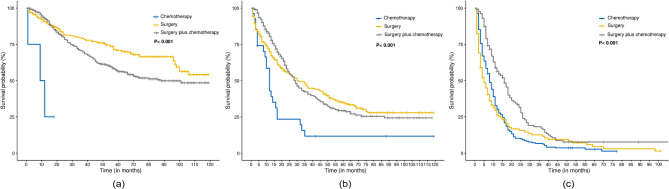
Kaplan‒Meier overall survival curves for treatment regimens in the three risk subgroups. Scores for the three subgroups ranged from (**A**) low risk: ≤ 206; (**B**) intermediate risk: (206, 282]; and (**C**) high risk: >282. Comparison of CSS in patients receiving chemotherapy alone, surgery alone, or surgery plus chemotherapy in the different risk groups

### Selection of treatment strategies based on nomograms

Overall, the 3-year CSS for patients with SBA was 47.2%, and the 5-year CSS was 36.9%. Based on the nomograms derived from the SEER database, SBA patients were categorized into three risk subgroups, each with different overall mortality risks. The prognostic outcomes of different treatment modalities were then compared within these risk subgroups. The treatment methods were categorized into three groups: chemotherapy, surgery, and surgery combined with chemotherapy.

In the low-risk group, there was a significant difference in survival rates among the three treatment methods (*P* < 0.001). Patients in this group may benefit from appropriate surgery alone. The combination of surgery and chemotherapy did not show a superior benefit compared with surgery alone. In the moderate-risk group, patients who underwent surgery alone had the best CSS (*P* < 0.001). Notably, patients in this group who received chemotherapy alone had the poorest prognosis in terms of survival; thus, organ-preserving treatments alone are not recommended for this group. In the high-risk group, significant differences in survival rates were observed among the three treatment methods (*P* < 0.001). The impact of surgery combined with chemotherapy was statistically significant (*P* < 0.001); thus, surgery combined with chemotherapy is recommended for these patients. It is important to note that patients who underwent surgery alone still had better CSS outcomes than those who received chemotherapy alone (*P* < 0.001). These findings emphasize the potential value of nomograms for tailoring effective treatment strategies based on individual risk assessments.

## Discussion

From an epidemiological standpoint, although SBA is still a relatively rare tumor, its annual incidence has been steadily increasing [17]. Our study highlights the complexities involved in predicting and improving the prognosis of patients with this rare but challenging form of cancer. These findings underscore the advantages of nomograms over traditional staging systems and emphasize the shift in clinical oncology toward personalized medicine. Reports suggest that nomograms provide greater predictive accuracy and prognostic value than existing tumor staging systems in many cancers [18–20]. Therefore, the development of an effective nomogram model is crucial for predicting survival in SBA patients and facilitating individualized treatment.

Although recent studies have developed prognostic models for SBA, factors such as adjuvant chemotherapy, radiotherapy, and regional lymph node dissection that influence the survival rates of SBA patients have been overlooked [21]. Compared with previous prognostic models, our research established the most comprehensive CSS nomogram for SBA patients. The C-index, ROC curves, calibration curves, DCA, NRI, and IDI all demonstrated that the nomogram possesses robust predictive capability and clinical applicability.

Multivariate analysis identified independent risk factors affecting the prognosis of patients with SBAs. In our study cohort, the proportion of male patients was greater than that of female patients (54.8% vs. 45.2%). Interestingly, female sex appears to be a protective factor against CSS in SBA patients. This is consistent with previous studies [22]. This may be because estrogen plays a role in the development and progression of cancer. Some studies have shown that postmenopausal women who use hormone replacement therapy (HRT) have a reduced risk of developing colorectal cancer. The proposed mechanisms include the effects of estrogen on bile acid metabolism, insulin sensitivity, and inflammation [23]. Thus, it may affect the prognosis of SBA patients. Advanced age is a significant prognostic factor for cancer outcomes. As shown in the survival plots, patients older than 70 years exhibit a significantly increased risk of mortality compared with those under 50 years, which is also supported by earlier research [8]. The mechanisms underlying this association may involve age-related factors such as diminished immune responses and elevated levels of chronic inflammation, which could impact the survival of SBA patients [24, 25].

Furthermore, this study indicated that being unmarried is associated with a poor prognosis in patients with SBA, which is consistent with previous findings [26]. However, another study on the metastatic patterns of SBA did not demonstrate this association [27]; this discrepancy may be associated with differences in the study populations. Unmarried patients were less likely to undergo surgery than were their married counterparts (55.9% vs. 66.0%, *P* < 0.001) [28]. Encouragement and financial support from the spouses of married patients may facilitate their acceptance of surgery and adjunctive therapies, partially explaining these disparities [29, 30].

Tumor location is also a critical factor in the prognosis of SBA patients. In our study cohort, more than half of the small intestine tumors were located in the duodenum (52.4%). In recent years, the incidence of SBA has been gradually increasing, primarily owing to the increasing incidence of duodenal cancer [31, 32]. This study demonstrated that, compared with jejunal sites, the duodenal location is a negative prognostic factor for patient survival, consistent with previous findings [33]. This may be because duodenal SBA patients typically present at more advanced stages, which is associated with delayed diagnosis and lower rates of tumor-related surgeries [34]. Moreover, duodenal cancer more readily invades nearby structures such as the pancreas, bile ducts, and mesenteric vessels. Some parts of the descending and horizontal duodenum are located retroperitoneally, where invaded lymph nodes can easily spread posteriorly, complicating surgical removal [35]. In addition, the duodenum is continuously exposed to bile and pancreatic enzymes, which have carcinogenic properties. These substances cause chronic irritation and inflammation of the mucosa, leading to DNA damage and increasing the likelihood of malignant transformation. Continued inflammation also leads to a constant cycle of cellular damage and repair, increasing the risk of mutation [36]. Inflammation also promotes an immunosuppressive environment that allows tumors to grow and spread. It is important to note that compared with jejunal tumors, duodenal tumors typically exhibit different genetic and molecular characteristics. For example, mutations in the APC gene, common in familial adenomatous polyposis (FAP), lead to the development of polyps into cancer, and alterations in signaling pathways such as the Wnt/β-catenin pathway and mutations in mismatch repair genes (as observed in Lynch syndrome) lead to aggressive cancerous behavior in duodenal adenocarcinomas [37–39].

Our survival plots underscore the significant contribution of pathological grade, consistent with prior studies that identified pathological grade as an independent predictor of survival [40, 41]. Poorly differentiated or undifferentiated tumors frequently exhibit malignant progression. This may be explained by the fact that tumors with lower pathological grades may have a tumor microenvironment that effectively evades the immune system. This may be due to high levels of immune checkpoint proteins (e.g., PD-L1), leading to immunosuppression and poor prognosis [42]. In addition, the presence of cancer stem cells in low grade differentiated tumors may lead to drug resistance and metastasis, resulting in a poorer prognosis [43]. Tumors with lower pathological grades may be more dependent on aerobic glycolysis (the Warburg effect), which can support rapid tumor growth and survival under hypoxic conditions [44]. Additionally, our model indicated that compared with stages T1–T2, N0, and M0, stages T3–T4, N1–N2, and M1 were negative prognostic factors for the survival of SBA patients. Previous studies have confirmed that T stage is an independent prognostic factor in SBA patients, with advanced T stage being associated with significantly lower survival rates [45, 46].

Our multivariate analysis of data from the SEER database indicated that patients with SBA who underwent regional lymph node dissection of ≥ 4 nodes during surgical treatment had a significantly greater survival rate than those who underwent removal of fewer or no nodes. This finding aligns with the hypothesis that more extensive lymph node dissection ensures more thorough elimination of the potential for metastasis, thereby improving the effectiveness of tumor treatment. Previous studies have similarly emphasized the importance of lymph node dissection in improving the prognosis of gastrointestinal malignancies. Choi et al. reported that patients with CRC who underwent extensive lymph node resection had significantly improved disease-free survival [47]. Although SBA is rare and there are few relevant studies, our findings suggest that the principles of adequate lymph node dissection might similarly apply. The correlation between the RORLN and survival outcomes may reflect the aggressiveness of SBA and the necessity for comprehensive surgical treatment. Our study supports the view that the number of lymph nodes dissected should be considered in surgical planning for SBA to maximize the chance of achieving favorable treatment outcomes for patients.

While some studies suggest that radiotherapy can also contribute to improving survival outcomes for patients with SBA [48, 49], the present analysis using SEER database data does not support this trend. A possible reason could be the potential biases in the current data on radiotherapy in the SEER database, given that many factors influencing treatment are not captured in the registry data. Radiotherapy is typically not the first-line treatment for SBA. Clinicians need to reassess the value of radiotherapy, as radiation can potentially harm the small intestine and surrounding tissues.

Currently, surgery remains the primary treatment option for patients with SBA, and curative surgery along with adequate lymph node dissection is critical for improving patient prognosis. Survival plots show that missing surgery significantly lowers survival rates; furthermore, risk-stratified Kaplan‒Meier curves demonstrated that surgery alone remains the preferred treatment method for low- and intermediate-risk groups, providing extended survival times. Furthermore, many past studies have demonstrated that adjuvant chemotherapy can significantly enhance overall survival (OS) and disease-free survival (DFS) [7, 50]. However, a retrospective study noted that adjuvant chemotherapy did not improve postoperative OS or DFS in SBA patients [51]. In our study, 53.4% of patients underwent chemotherapy, with those receiving chemotherapy showing a significantly reduced survival risk (HR = 0.52, *P* < 0.001), indicating that chemotherapy plays a positive role in improving the prognosis of SBA patients. Intuitive survival plots can also be used to encourage patients with SBA to actively pursue treatment. In high-risk patients, combining surgery with chemotherapy can increase patient survival. Palliative surgery for some patients with advanced SBA who have complications such as intestinal obstruction and intestinal bleeding can relieve the obstruction and stop the bleeding, thus relieving the symptoms and improving the patients’ quality of life. Palliative surgery can also reduce the tumor load in the body and create favorable conditions for subsequent chemotherapy. Unfortunately, the SEER database does not provide specific chemotherapy regimens or drug selections, precluding subgroup analysis. We look forward to future updates of the database that may provide this information.

Compared to the AJCC staging system, the survival plot model demonstrated better predictive capability for survival. Despite these contributions, our study has several limitations. First, it utilizes information from the SEER database for statistical analysis, and as a retrospective study, it is inherently biased, necessitating future verification through prospective research. Second, we excluded patients whose information was incomplete, which could have led to selection bias. Moreover, the study did not include several important factors, such as tumor markers, body mass index (BMI), immunohistochemical markers, or genetic mutation status. Although these factors are missing from the SEER database, they may be related to the prognosis of SBA patients. Finally, the survival plot model has only been internally validated using the database, and although it performed well, it is still necessary to evaluate the model’s accuracy through external validation in other populations.

## Conclusion

In conclusion, we developed and validated a survival plot model to predict the 3-year and 5-year CSS rates of patients with rare SBA. The model demonstrates good discriminative ability and calibration, and its simplicity makes it a convenient tool. Compared to the AJCC staging system, it shows superior survival prediction capabilities. Survival plots may assist clinicians in predicting individual patient outcomes and offering improved treatment recommendations. However, future studies will require further multicenter external validation to obtain more convincing and directive results.

## Data Availability

The original contributions presented in the study are included in the article, and further inquiries can be directed to the corresponding author.
